# Integrating symptom science and precision health to enhance palliative care outcomes

**DOI:** 10.3389/fmed.2026.1870146

**Published:** 2026-08-05

**Authors:** Taichi Goto, Judy Barberio, Leorey N. Saligan

**Affiliations:** 1 Department of Research, The Cizik School of Nursing, The University of Texas Health Science Center at Houston, Houston, TX, United States; 2 Division of Nursing Science, Rutgers School of Nursing, The State University of New Jersey, New Brunswick, NJ, United States

**Keywords:** biomarkers, digital health, multi-omics, palliative care, precision health, psychoneurological symptoms, symptom science

## Abstract

**Background:**

Precision health is transforming the landscape of palliative care by enabling more individualized approaches to symptom assessment and management. Symptoms remain the primary focus of palliative care; however, traditional approaches rely largely on subjective self-report and often fail to capture the biological mechanisms underlying symptom variability and clustering.

**Objective:**

This paper examines how integrating symptom science with precision health approaches, including multi-omics, digital health technologies, and systems biology, can enhance symptom characterization and inform individualized palliative care interventions.

**Methods:**

This perspective paper focuses translational and clinical evidence, including exemplars from the authors’ program of research utilizing genomic, metabolomic, inflammatory, and extracellular vesicle biomarkers to characterize psychoneurological symptom clusters in individuals with serious illness. Supporting literature on digital health integration and ethical considerations in palliative care was also reviewed.

**Results:**

Emerging evidence demonstrates that multi-omic approaches may help identify biological mechanisms underlying symptom heterogeneity and clustering, enabling more precise phenotyping and informing the development of targeted interventions. Genotype–phenotype associations, such as those involving the brain-derived neurotrophic factor (BDNF) *Val66Met* polymorphism, and biomarker-informed symptom clusters, highlight opportunities for the future development of mechanism-based symptom management strategies. Digital health interventions, including remote symptom monitoring and telehealth, have demonstrated improvements in symptom burden, quality of life, and care coordination although cost-effectiveness may vary across technologies, settings, and healthcare systems. However, variability in biological pathways and challenges related to implementation, equity, and access underscore the need for thoughtful integration.

**Innovation:**

This paper is innovative in its integration of multi-omic symptom science with precision health frameworks to advance a mechanism-informed model of palliative care. It uniquely bridges biological, technological, and clinical domains to move beyond symptom description toward biologically grounded, individualized symptom management, while explicitly preserving the “high-tech, high-touch” ethos central to palliative care.

**Conclusion:**

Integrating precision health strategies with symptom science offers a transformative opportunity to advance palliative care through individualized, mechanism-informed symptom management. A “high-tech, high-touch” approach is essential to ensure that technological innovation enhances, rather than replaces, the compassionate, person-centered care that defines palliative practice. Future directions should prioritize scalable models, non-invasive biomarkers, and equitable implementation across diverse populations.

## Introduction

**FIGURE 1:**
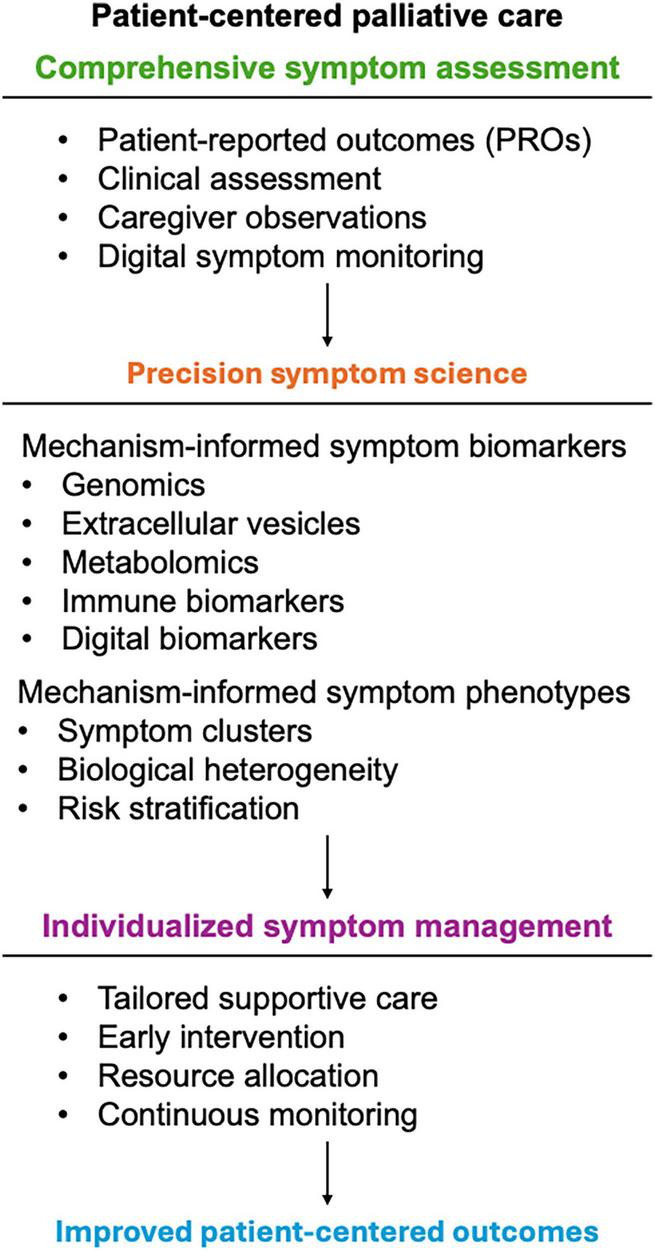
Conceptual framework integrating symptom science and precision health to advance patient-centered palliative care. Comprehensive symptom assessment, combined with precision symptom science, identifies mechanism-informed symptom phenotypes and supports individualized symptom management while maintaining patient-centered outcomes.

Precision health is an emerging approach to disease treatment and prevention that accounts for individual variability in disease experiences (1). Within this framework, symptom science is a key domain wherein precision health strategies are used to improve outcomes for individuals with serious or chronic illness. Symptoms are self-reported perceptions (2) that have clinical, research, and policy relevance because they are the most common reason that patients seek healthcare (3). In palliative care, symptoms are the primary targets of assessment and management, as they directly impact patients’ quality of life, functional status, and overall well-being, and they are central to alleviating suffering (4, 5). Understanding the etiology of symptoms is a key scientific priority in advancing palliative care and represents an area where precision health strategies can have the greatest impact (Figure 1).

New advances in precision health offer the potential to more precisely characterize symptom profiles and clusters, to inform more targeted symptom assessment and intervention strategies, with direct implications for palliative care. For instance, advances in laboratory sciences (e.g., omics approaches), technological sciences, and big data management have enabled scientists to better phenotype symptoms and symptom clusters associated with health and illness. Similarly, “omics” approaches have paved the way to improved understanding of the influences of biomolecules in the development or worsening of symptoms (6, 7) and have contributed to the development of personalized interventions and precision health care (8). Technological advancements have also enabled innovative tools to customize symptom management (9). Perspectives in systems biology applied to mathematical metaparadigms enable scientists to organize and manipulate new and unique types of big data, using greater computational capacities to conceptualize health outcomes (10). Thus, by integrating omics, technological innovations, and systems biology approaches, clinicians and researchers may improve their ability to predict symptom trajectories and tailor symptom management strategies to individual patients, with the potential to enhance quality of life for those with serious or life-limiting illnesses. Although the exemplars discussed in this paper focus primarily on the biological and technological dimensions of symptom science, we recognize that symptom experiences in palliative care are shaped by the interaction of biological, psychological, social, and existential factors.

## Patient-centered and compassionate approaches to palliative care

Symptom relief is one of six concepts in the 6S-model for person-centered palliative care, which also includes Self-image, Self-determination, Social relationships, Synthesis, and Strategies (11). The model emphasizes that high-quality palliative care should address not only physical symptoms but also the psychological, social, and existential dimensions of a person’s experience as illness progresses and death approaches (11). As precision health advancements evolve and are integrated into treatment for serious or life-limiting illnesses, it remains essential to maintain the “high-touch” relational care that defines palliative practice (12). The central ethical imperative is to uphold a “high-touch within high-tech” model of care, ensuring that technological innovation enhances, rather than erodes, the personalized, values-concordant relationships that patients and families regard as essential. Effective implementation requires that precision-based tools support communication, anticipate clinical needs, and optimize symptom management while safeguarding the individualized, culturally attuned assessments that define high-quality palliative care.

Weaver et al. reported a telehealth-supported hospice model in which a familiar pediatric palliative care provider joined home-based visits in rural settings (13). By linking a hospital-based palliative care physician already known to the family with a rural home hospice nurse, the intervention supported caregiver needs, symptom management, goals of care, and medication review while extending continuity of palliative care into the home. Harris et al. also evaluated an online tool that enabled families to identify, prioritize, and monitor issues affecting their quality of life (14). The authors reported that the tool improved family empowerment and supported communication between families and professionals, thereby supporting a more patient- and family-centered approach to palliative care.

## Exemplar of a multi-omic symptom science approach

### From self-report to mechanism-informed precision symptom care

Symptom assessment in serious illness and chronic conditions has relied primarily on patient self-report, which remains essential but does not capture underlying mechanisms or biological heterogeneity. Over the past decade, our group and collaborators have operationalized this model in people with cancer and other serious illnesses, particularly focusing on psychoneurological (PN) symptoms such as fatigue, pain, sleep disturbance, mood symptoms, and cancer-related cognitive impairment (CRCI). These studies have used multi-omic platforms, including mitochondrial and metabolic profiling, immune and extracellular vesicle (EV) markers, gene and pathway-level analyses, and candidate polymorphisms such as the brain-derived neurotrophic factor (BDNF) *Val66Met* variant. The following exemplars from our research program illustrate how mechanism-focused approaches can inform precision symptom science in palliative care.

### Exemplar 1: brain-derived neurotrophic factor (BDNF) and psychoneurological symptom burden

Cancer survivors frequently experience persistent PN symptoms even after completing cancer treatment. Although prior work has linked cancer and its therapies to specific symptom phenotypes, these factors alone do not fully explain the marked inter-individual variability and heterogeneity in PN symptoms. Recent evidence suggests that single-nucleotide polymorphisms (SNPs) contribute to this variability. BDNF, a neurotrophin critical for neuronal survival and synaptic plasticity, has a common functional SNP, *Val66Met* (*rs6265*), which has been associated with PN symptoms, including anxiety, depression, and cognitive impairment, with mixed findings (15–18). Accordingly, our program of research has focused on clarifying the genotype–phenotype relationships between *Val66Met* and cancer-related PN symptom burden.

In male cancer survivors, most of whom had non-metastatic prostate cancer (NMPC), we found that *Met* allele carriers (*Met/Met* and *Val/Met*) reported significantly lower self-reported fatigue levels than *Val/Val* wild-type homozygotes, suggesting that the *Met* allele may confer reduced risk for cancer-related fatigue in this population (19). This pattern was supported by our preclinical work: transgenic mice homozygous for the human *Val66Met* variant exhibited reduced fatigue-like behavior after 5-fluorouracil administration compared to wild-type mice (20). In contrast, in our cohort of female cancer survivors, the majority with breast cancer, *Met/Met* carriers reported more severe self-reported fatigue and neuropathic pain than those with the *Val/Val* genotype (21). Taken together, these findings suggest a potential sex-specific difference in the relationship between the *BDNF Val66Met* genotype and fatigue-related phenotypes, warranting further investigation that accounts for sex as a biological variable.

Cancer-related cognitive impairment is also a major problem in cancer survivors. In our work with breast cancer survivors, those with the *BDNF Val66Met Met*/*Met* genotype demonstrated poorer performance on objectively assessed visual episodic memory/new learning and spatial working memory/executive function, even after adjusting for age and other PN symptoms as potential confounders (22). We further examined CRCI in relation to combined genotypes for *BDNF rs6265* and apolipoprotein E (*APOE*) *rs7412*. Survivors carrying the *APOE Arg*/*Cys* genotype together with *BDNF Val*/*Val* or *Val*/*Met* showed better cognitive performance than other genotype combinations, suggesting that direct or indirect interactions between these SNPs may influence cognitive function phenotypes in cancer survivors (23).

### Exemplar 2: systemic inflammation, extracellular vesicles, metabolomics, and symptom clusters

Psychoneurological symptoms frequently co-occur and form symptom clusters. In patients with fibromyalgia, we identified two distinct clinical subclusters: one characterized by widespread pain, unrefreshed waking, and somatic symptoms, and a second characterized by prominent fatigue and cognitive dysfunction, with pain that was less severe and less widespread (24). Among cancer survivors receiving radiotherapy for NMPC, a multivariable regression model showed that 74% of the variance in persistent fatigue at 1 year was explained by urinary dysfunction, pain, and depressive symptoms, indicating that fatigue is embedded within a broader co-occurring symptom constellation rather than occurring in isolation (25). In community-dwelling older adults with chronic conditions, we further identified three symptom clusters: high symptom burden, pain and fatigue, and sleep deprivation, with the high burden group more likely to be older, female, living in rural areas, physically inactive, and living with multiple chronic conditions (26). Collectively, these findings suggest that recurring configurations of pain, fatigue, sleep disturbance, mood, and functional problems may reflect shared underlying biological mechanisms that drive symptom clustering across diagnoses and care settings.

Therefore, we next examined how PN symptom clusters relate to extracellular vesicle (EV)-derived and blood-based soluble biomarkers. EVs are membrane-enclosed particles, delimited by a phospholipid bilayer and spanning the nano- to micron-size range, and a growing body of evidence indicates that they possess specialized functions and play key roles in a variety of physiological and pathological processes (27). In an exploratory study of men with NMPC receiving external beam radiation therapy, we found that patients who developed worsening cancer-related fatigue over 3 months showed greater increases in EV-associated Eotaxin, HSP27, IP-10, and MIP-3α, and that both EV-associated (e.g., CRP, MCP-1) and soluble markers (e.g., survivin, IFNα2, IL-8, IL-12p70, MCP-1) were significantly correlated with lower FACT-F scores (worse fatigue) (28). Building on this, we used principal component analysis to identify PN symptom clusters. At the start of radiation therapy, a PN cluster comprising cancer-related fatigue, cognitive fatigue, and sleep disturbance was associated with multiple EV-associated cytokines and growth factors, including IL-1α, IL-15, IL-21, GM-CSF, M-CSF, and RANTES, together with soluble RANTES, IL-21, and IL-33. By 3 months after completing radiation therapy, however, the PN symptom cluster, which now also included depressive symptoms, was no longer correlated with EV-derived markers but instead showed positive associations with soluble IFNα2, IL-9, and IL-17 (29). These temporal shifts suggest that radiation therapy-induced biological alterations may change not only the intensity and composition of PN symptom clusters but also the inflammatory compartments (EV-associated versus soluble) and specific cytokine pathways that track with these symptom phenotypes.

Metabolomics, enabled by ultrahigh-performance liquid chromatography–mass spectrometry (UHPLC/MS), allows comprehensive identification and quantification of small-molecule metabolites in biofluids and provides a real-time functional readout of biochemical activity and cellular state (30). Using an untargeted UHPLC/MS approach, we profiled 1,120 plasma metabolites in fatigued cancer survivors, non-fatigued cancer survivors, and healthy individuals classified by the Functional Assessment of Chronic Illness Therapy-Fatigue (FACIT-F) (31). Partial least squares discriminant analysis demonstrated clear separation of metabolomic profiles among the three groups, and KEGG pathway enrichment analysis revealed that pathways uniquely overrepresented in fatigued cancer compared with controls included sphingolipid metabolism, histidine metabolism, and cysteine and methionine metabolism. Together, these findings begin to define a metabolic phenotype of cancer-related fatigue that could be leveraged to develop candidate mechanism-based biomarkers and inform future targeted interventions.

### Implications of the multi-omics and biomarker-based approaches to precision palliative care

Symptom assessment at the end of life still relies largely on self-report and verbal communication, reflecting the inherently personal and heterogeneous nature of symptom experiences. However, patients receiving palliative care are often less able to communicate reliably because of cognitive impairment, delirium, language barriers, or overall clinical decline (32). There is, therefore, a critical need for objective biomarkers that nevertheless capture, or closely mirror, patients’ lived symptom experiences. While our prior work has focused primarily on blood-derived EVs and metabolites, these analytes are also present in other biofluids, such as urine and saliva, which can be obtained non-invasively (27, 31). In combination with genotype-based identification of vulnerable subgroups, non-invasive quantification of EV- and metabolite-based biomarkers has the potential to enable more precise symptom monitoring and inform symptom management strategies, with the goal of improving patient-centered outcomes in palliative care settings.

The approaches discussed in this paper may have relevance across the entire cancer continuum. During active treatment, biomarker-informed symptom monitoring may facilitate earlier identification of patients at risk for severe cancer-related symptoms, including fatigue and cognitive impairment (19, 22, 28), thereby enabling more timely supportive care interventions. In cancer survivorship, multidimensional strategies that integrate multi-omics, digital health technologies, and social determinants of health may help identify individuals at risk for persistent symptom burden and functional decline (26, 31). In advanced illness and home-based palliative care, combining biologic, behavioral, and digital health data may support proactive symptom management, timely care coordination, and anticipatory decision-making as patients approach the end of life (12, 33).

### Limitations and challenges of the multi-omics and biomarker-based approaches

These exemplars highlight the potential of multi-omic and biomarker-based approaches to support more individualized symptom assessment and management in palliative care settings. However, several limitations and challenges still need to be addressed before these approaches can be translated into routine clinical practice.

First, many prior studies have used exploratory designs with relatively small sample sizes. For example, Sass et al. examined associations between EV-derived biomarkers and cancer-related symptoms in men with non-metastatic prostate cancer, but also noted that multiple-comparison correction was not performed because of the small sample size (*n* = 40) and the exploratory nature of the studies, which may have increased the risk of both type I and type II statistical errors (28, 29). Several studies investigating the relationships between SNPs and cancer-related symptoms were also hypothesis-generating or secondary analyses, and some did not perform *a priori* power analyses (21–23). Moreover, these studies were conducted largely in White cohorts (21–23), even though the frequency of the *BDNF rs6265 Met* allele differs across racial and ethnic populations (19, 21–23). Findings from relatively homogeneous samples may therefore have limited generalizability, particularly when the biomarkers of interest themselves vary across populations.

In addition, many of these studies have focused on a single disease context, such as breast cancer or non-metastatic prostate cancer (19, 21–23, 28, 29). As a result, it remains unclear how well findings from one study population can be generalized to other cancers, non-cancer serious illnesses, or broader palliative care populations. Many of these investigations should therefore be viewed as hypothesis-generating and will require further evaluation for reliability, validity, reproducibility, and external validation in independent, larger, and more diverse cohorts, as well as in translational and preclinical studies designed to clarify mechanisms.

Multi-omic platforms also generate large, high-dimensional datasets that require sophisticated laboratory methods, bioinformatic infrastructure, and advanced analytic expertise. The cost and complexity of implementing such workflows may limit their feasibility in routine clinical settings. In addition, there remains a substantial gap between biomarker discovery and the development of clinically feasible interventions that can meaningfully reduce symptom burden in palliative care. Researchers must therefore remain attentive to these limitations and ensure that multi-omic discovery ultimately directs toward actionable, patient-centered symptom management.

## Real-world implementation and ethical considerations

Such recent precision health advances, though promising for palliative care, also raise critical questions about how these approaches can be implemented feasibly, proportionately, and equitably in real-world palliative care settings, and how to ensure that such innovations do not erode the person-centered, compassionate ethos of palliative care.

Workforce shortages are already a major challenge in palliative care. In the United States, palliative care needs have been estimated to exceed specialist workforce capacity by approximately 1.8- to 3.3-fold, and these shortages are expected to persist or worsen over time (34, 35). Implementing multi-omic and biomarker-based approaches, which may require additional personnel and training in molecular, computational, and bioinformatic methods, could further strain already limited clinical resources (12, 36). In addition, patients and caregivers may face barriers related to digital access, proficiency, and willingness to use monitoring technologies. Viana et al. noted that older adults may be reluctant to engage with digital platforms, while younger patients may also be hesitant to be monitored by health care providers, suggesting that variation in digital literacy and acceptability may limit participation and real-world uptake (12).

While the significant costs associated with multi-omics technologies and their cost-effectiveness remain important challenges for clinical implementation (37), the long-term economic impact of precision health approaches is still evolving. Cost-effectiveness analyses of comprehensive genomic profiling have demonstrated improvements in quality-adjusted survival but have not consistently shown favorable cost-effectiveness ratios under current pricing structures (37). Conversely, some observational studies suggest that precision oncology approaches may be associated with lower healthcare utilization and reduced end-of-life costs, potentially offsetting part of the upfront investment in genomic testing and targeted therapies (38). These findings suggest that, under some circumstances, reductions in healthcare utilization and end-of-life costs may partially offset the upfront costs of genomic testing and targeted therapies. However, additional prospective health economic evaluations are needed to determine whether such benefits are generalizable across healthcare settings and patient populations.

Technical and organizational barriers also complicate implementation. Integrating multi-omic data with electronic health records remains difficult because of heterogeneous data sources, lack of interoperability, and nonstandardized genomic reporting formats (36, 39). In addition, omics-based analyses may be time-consuming and computationally demanding, which limits their practicality in late-stage palliative care settings where timely decision making is essential (39). High costs related not only to data acquisition, but also to storage, computation, cybersecurity, and infrastructure maintenance, further challenge feasibility at the health-system level (12, 36).

The proportionality of these approaches also requires careful consideration. Although noninvasive biospecimen collection may sometimes be possible, many biomarker and multi-omic approaches still rely on repeated blood sampling and ongoing monitoring. In frail patients, those with cognitive impairment, or those near the end of life, the burden of repeated testing and data collection may outweigh potential benefit, particularly when findings are not immediately actionable (12). Precision approaches in palliative care should therefore be implemented only when they are likely to inform care in a meaningful way. Genomic testing may also have implications for biologic relatives, creating a need for appropriate support services such as genetic counseling (12).

Equity is another major consideration. Existing disparities in access to palliative care and digital health infrastructure mean that the benefits of precision health are unlikely to be distributed evenly. Patients receiving palliative care are more likely to be White, socioeconomically advantaged, and treated in academic or resource-rich settings, while digital exclusion, geography, and underrepresentation in omics datasets may further widen inequities (12, 35). These concerns underscore the need for inclusive technology development, diverse validation cohorts, and implementation strategies that are responsive to local resources and patient populations (12).

Ethical considerations are another essential dimension of real-world implementation. Viana et al. (12) note that while advancements in integrated systems biology and advanced digital technologies – such as multi-omics analyses and continuous health monitoring platforms – can deepen insight into biologic trajectory and symptom progression in seriously ill patients, they also raise important ethical, equity, and relational questions: How can we ensure that high-tech modalities do not undermine the core “high-touch” ethos of palliative care? How do we include marginalized and underrepresented populations in both the development and deployment of these technologies? And what are the implications of prognostic algorithms on patient autonomy and care decision-making? Drawing on these reflections, the authors propose the following ten recommendations aimed at ethically, inclusively, and equitably integrating precision health tools into palliative care settings, ensuring that innovation enhances rather than erodes the person-centered, compassionate ethos of palliative care.

1. **Patients’ goals, values, and lived symptom experiences should remain central to precision palliative care.**
  Precision health approaches should be used to clarify and support patient-defined priorities and to relieve suffering, rather than allowing technology itself to become the focus of care. This is especially important in palliative care, where patients with the same diagnosis or disease stage may have markedly different symptom burden, coping capacity, family needs, and goals of care. Sedhom et al. argue that a one-size-fits-all model of early palliative care may misallocate limited specialist resources because referral based only on disease status can overlook patients with the greatest supportive care needs while directing services to others with little immediate need (35). Individualized, precision care may allocate limited resources to appropriate individuals, thereby alleviating the workforce shortage (35). This emphasis is consistent with the National Consensus Project guidelines, which state that palliative care begins with a comprehensive assessment and requires individualized care planning grounded in patient and family engagement, communication, care coordination, and continuity across settings (40).
2. **Palliative care systems should be designed to promote equity and consistent access to high-quality palliative care.**
  Technologies should be developed, deployed, and implemented in ways that reduce disparities and ensure that patients across populations and settings can benefit from comparable standards of symptom assessment and management. The introduction of precision and digital health technologies may otherwise exacerbate existing inequities related to race, socioeconomic status, language, and geography (12, 41). In the United States, Medicare beneficiaries, particularly older adults, racial and ethnic minority groups, and those living below the poverty level, are more likely to lack digital access at home, limiting their ability to benefit from technology-enabled care (12). Thus, these technologies and systems should be designed to meet the needs of underrepresented populations, taking into account socioeconomic, racial, cultural, and linguistic factors (12, 42). In addition, developers, researchers, and clinicians should engage in ongoing critical reflection on how structural inequities and implicit bias may shape technology development, data interpretation, and clinical decision-making (42).
3. **Human-centered and participatory design processes should involve patients, caregivers, and interprofessional providers from the outset.**
  Co-design helps ensure that digital and biologic technologies remain relevant, acceptable, feasible, and aligned with real-world needs and capabilities. Limited end-user involvement is a major reason why digital health interventions often fail to achieve sustained adoption (43). In palliative care, Al-Mondhiry et al. used community-partnered participatory research to co-design and pretest a mobile app for patients with advanced cancer, identifying priorities shared by clinicians and patients, including symptom management, communication support, access to vetted information, and patient advocacy (44). These findings suggest that early stakeholder involvement can improve the clinical relevance and usability of precision supportive care tools.
4. **Clinicians and care teams should be prepared through training in precision data literacy, communication, and ethical implementation.**
  Successful integration depends on workforce readiness to understand data limitations, communicate uncertainty, and incorporate findings into compassionate, person-centered care. Schaibley et al. found that only 20% of surveyed physicians reported moderate or extensive training in genetics, and lack of training in selecting appropriate tests and interpreting results was the most frequently reported barrier to clinical use (45). Mitchell et al. similarly reported that although frontline healthcare professionals recognized the potential of precision medicine, they also expressed concerns about limited infrastructure, lack of time for training, and uncertainty about how to use it in practice (46). Together, these findings suggest that implementation will require practical training that supports both technical competence and person-centered communication.
5. **Multi-omics approaches and digital health technologies should be applied selectively and proportionately to patient needs, clinical relevance, and informed preferences.**
  Because these technologies may generate unnecessary or unwanted information, their use should be guided by the patient’s condition, goals, and wishes. Méndez Hernández and Ramasco Rueda noted that an ideal biomarker should be easy to measure, low in cost, and high in sensitivity and specificity, while also providing added value beyond routine clinical assessment (47). They further emphasized that biomarkers should be evaluated for proof of concept, prospective validity, incremental value, clinical utility, improved outcomes, and cost-effectiveness before being integrated into practice (47). At the same time, routine symptom assessment through patient-reported outcomes remains essential for identifying patient and caregiver needs and for targeting referrals appropriately (35). Together, these principles support a selective and proportionate use of multi-omics and digital health technologies in palliative care.
6. **Clear safeguards for consent, privacy, data stewardship, and responsible data use should be established.**
  The collection, storage, sharing, and secondary use of sensitive biologic and digital data require explicit ethical oversight, transparency, and accountability. While genomic and other multi-omic data may directly inform patient care and improve outcomes, they also have substantial value for future research and knowledge generation (48). However, consent obtained for clinical purposes may not always be sufficient for secondary research use, and additional or renewed consent processes may be required (48). These considerations may be particularly important in palliative care, where patients may experience cognitive impairment, fluctuating decision-making capacity, or progressive clinical decline (12). Dynamic consent models, which allow individuals to monitor how their data are used and modify or withdraw consent over time, have been proposed as one approach to support patient autonomy as goals and preferences evolve (12, 48). Privacy protection is equally important. Even when direct identifiers are removed, genomic data remain potentially identifiable, and re-identification or membership inference attacks may be possible through linkage with publicly available databases (48). These risks underscore the importance of robust data stewardship, including oversight by data access committees, transparent governance frameworks, and clearly defined responsibilities for data storage, access, and sharing (12, 48). As precision palliative care increasingly incorporates prognostic algorithms, remote monitoring, and multi-omic testing, ethical implementation will require that these technologies support, rather than replace, shared decision-making among patients, families, and care teams.
7. **High-tech assessments should be used to identify individual patient characteristics and track within-person changes, while maintaining an active in-person presence to contextualize data.**
  The value of precision approaches lies not only in distinguishing differences between patients but also in capturing evolving symptom trajectories within the same patient, thereby enabling more personalized care. In a prospective study of continuous vital sign monitoring in a palliative care unit, heart rate increased as death approached and was positively correlated with pain severity, suggesting that continuous monitoring may detect clinically meaningful changes that could be missed by intermittent assessments (49). However, physiologic signals alone cannot fully capture patient needs. Routine patient-reported outcomes remain essential for identifying symptom burden, supportive care needs, and changing goals of care, enabling more timely and targeted interventions (35). Emerging approaches that integrate multi-omic data, digital health monitoring, and artificial intelligence (AI) may further improve the ability to identify deterioration and anticipate supportive care needs (12). Nevertheless, these technologies should complement ongoing conversations and in-person assessments that provide the clinical and relational context necessary for person-centered palliative care.
8. **Precision tools should be integrated into routine workflows in ways that strengthen multidisciplinary communication, coordination, and symptom management.**
  Technology should be implemented as a practical clinical support that enhances team-based care rather than adding fragmentation or replacing essential human interactions. Effective communication is particularly important in palliative care, where patients often have complex physical, psychosocial, and spiritual needs that require input from multiple disciplines (50). The quality of communication can directly influence patient outcomes and family satisfaction, while communication failures may contribute to care fragmentation, medication errors, and inconsistent implementation of care goals (50). Precision health technologies, including electronic health records, patient-reported outcomes, remote monitoring platforms, and multi-omic data systems, may facilitate real-time information sharing and coordinated decision-making across care settings (12). To be clinically useful, however, these tools should be embedded within existing workflows and support multidisciplinary collaboration rather than adding complexity or administrative burden (12, 50).
9. **Precision data should be interpreted cautiously and within their biological, technical, and clinical context.**
  Digital, biomarker, and omics data should never be treated as self-evident; clinicians must consider technical, biological, environmental, and clinical sources of variability when drawing conclusions. Omics and biomarker findings are influenced by multiple sources of variability, including sample collection procedures, sample quality, analytical methods, and batch effects, all of which can affect interpretation and reproducibility (28, 29, 31). Interpretation may also be complicated by population heterogeneity. For example, studies of the *BDNF rs6265* polymorphism have demonstrated different associations with symptom phenotypes across cohorts and suggest possible sex-related differences in genotype–phenotype relationships (19, 21, 22). AI and machine learning models introduce additional challenges because their recommendations may be influenced by underlying biases in training data, limited generalizability across populations and settings, and reasoning processes that are not always transparent to end users (51). Precision health data should be viewed as one component of a patient’s lived experience rather than reducing individuals to algorithmic predictions, probabilities, or percentages (12). Clinical judgment, patient narratives, and shared decision-making, therefore, remain essential when interpreting precision health findings and incorporating them into palliative care.
10. **A high-touch within high-tech model should be maintained, with human clinicians retaining final responsibility for judgment and care decisions.**
  Precision tools should support, not replace, the relational, ethical, and decision-making roles of clinicians and multidisciplinary teams. Human in the loop AI has emerged as a collaborative paradigm in which AI-based pattern recognition is combined with human contextual understanding, ethical reasoning, and clinical judgment (52). Evidence suggests that such approaches can improve diagnostic accuracy, reduce medical errors, enhance patient safety, and increase clinician trust when compared with either fully automated systems or clinician-only workflows (52). However, human oversight alone does not guarantee better decisions. Experimental evidence suggests that while human-in-the-loop designs increase acceptance of algorithmic recommendations, they may also introduce automation bias and do not necessarily improve decision accuracy (53).
  Therefore, efficiency gained through high-tech tools should not come at the expense of patient-centered, relationship-based care. Rather, these technologies should support clinicians in delivering more responsive, compassionate, and individualized care while preserving shared decision-making and professional accountability (12).

## Evidence for successful integration

Digital health technologies have been integrated into palliative care through models that enhance, rather than supplant, human connection. Across multiple studies, these tools have demonstrated improvements in symptom management, care coordination, and patient-reported outcomes while maintaining the field’s person-centered ethos.

Electronic symptom monitoring shows particular promise in home-based palliative care, with documented improvements in quality of life, physical and emotional well-being, and symptom burden, while available evidence suggests that these benefits may be achieved without a significant increase in costs (54). A meta-analysis reported that digital health interventions significantly improved overall symptoms [standardized mean difference (SMD) = −0.21] and reduced pain intensity (SMD = −0.49) among patients with advanced cancer (55). An umbrella review of 25 systematic reviews further concluded that digital health interventions were consistently beneficial or noninferior to standard care across multiple outcomes, with no reported adverse effects (56).

Digital technologies have also strengthened care coordination and communication, particularly in outpatient settings where spatial and temporal barriers often impede timely care (57). These tools support ongoing symptom monitoring, care coordination, and patient–provider communication, while preserving the essential contributions of multidisciplinary teams (58).

## Preserving core values while advancing care

Clinicians generally endorse digital technologies when they augment, rather than replace, human interaction (57). Providers emphasize that mobile health tools should “facilitate palliative care rather than replace important multidisciplinary services,” noting concerns about depersonalized assessment and care when technology is overused (58).

Human-centered design has emerged as a critical strategy for developing meaningful digital solutions. Participatory approaches that incorporate the perspectives of patients, caregivers, and interprofessional providers help ensure that technologies remain relevant and acceptable to all stakeholders (59). Effective implementation requires attention to language, context, and delivery; alignment with individualized health goals; support for family and caregivers; and sensitivity to older adults’ technological abilities (58).

## Future directions

To integrate precision health advances while preserving palliative care’s core values, several strategies are recommended. Digital tools and AI should be deployed in complementary roles, supporting mortality prediction, symptom monitoring, needs identification, and resource allocation, while reserving complex decision-making and emotional support for human clinicians (60). Although digital symptom monitoring interventions have demonstrated benefits for several patient-reported outcomes, evidence specifically supporting AI-driven symptom monitoring remains limited (61). Additional prospective trials are needed to determine whether AI-based approaches provide incremental benefits beyond standard care with respect to quality of life, symptom burden, healthcare utilization, and cost-effectiveness.

Ethical and equity considerations require ongoing oversight, inclusive development processes that engage underrepresented and marginalized groups, and careful evaluation of how technology influences therapeutic relationships (12). Barriers such as technical challenges, organizational constraints, resource limitations, and reduced nonverbal communication must be addressed through multifaceted strategies that combine technological enhancements, workforce training, organizational commitment, ethical safeguards, and equitable access (56).

Workflow integration is equally essential. Evidence from German healthcare professionals indicates that digital technologies already facilitate coordination of work processes, patient-centered care, and communication–contributing to more efficient, responsive, and effective palliative care when thoughtfully implemented (57).

Collectively, the evidence suggests that precision health tools can meaningfully advance palliative care when developed and deployed with explicit attention to preserving the relational, compassionate foundation that defines the field. Technology is most effective when used to amplify, not replace, the human elements of care that patients and families value most.
